# Transformations of sensory information in the brain suggest changing criteria for optimality

**DOI:** 10.1371/journal.pcbi.1011783

**Published:** 2024-01-11

**Authors:** Tyler S. Manning, Emma Alexander, Bruce G. Cumming, Gregory C. DeAngelis, Xin Huang, Emily A. Cooper

**Affiliations:** 1 Herbert Wertheim School of Optometry & Vision Science, University of California, Berkeley; 2 Department of Computer Science, Northwestern University, Illinois, United States of America; 3 Laboratory of Sensorimotor Research, National Eye Institute, National Institutes of Health, Maryland, United States of America; 4 Department of Brain and Cognitive Sciences, University of Rochester, New York, United States of America; 5 Department of Neuroscience, University of Wisconsin, Madison; 6 Helen Wills Neuroscience Institute, University of California, Berkeley; UT Austin: The University of Texas at Austin, UNITED STATES

## Abstract

Neurons throughout the brain modulate their firing rate lawfully in response to sensory input. Theories of neural computation posit that these modulations reflect the outcome of a constrained optimization in which neurons aim to robustly and efficiently represent sensory information. Our understanding of how this optimization varies across different areas in the brain, however, is still in its infancy. Here, we show that neural sensory responses transform along the dorsal stream of the visual system in a manner consistent with a transition from optimizing for information preservation towards optimizing for perceptual discrimination. Focusing on the representation of binocular disparities—the slight differences in the retinal images of the two eyes—we re-analyze measurements characterizing neuronal tuning curves in brain areas V1, V2, and MT (middle temporal) in the macaque monkey. We compare these to measurements of the statistics of binocular disparity typically encountered during natural behaviors using a Fisher Information framework. The differences in tuning curve characteristics across areas are consistent with a shift in optimization goals: V1 and V2 population-level responses are more consistent with maximizing the information encoded about naturally occurring binocular disparities, while MT responses shift towards maximizing the ability to support disparity discrimination. We find that a change towards tuning curves preferring larger disparities is a key driver of this shift. These results provide new insight into previously-identified differences between disparity-selective areas of cortex and suggest these differences play an important role in supporting visually-guided behavior. Our findings emphasize the need to consider not just information preservation and neural resources, but also relevance to behavior, when assessing the optimality of neural codes.

## Author summary

The nervous system needs to transform information from the sensory organs into signals that can be used to guide behavior. Neural activity is noisy and can consume large amount of energy, so sensory neurons must optimize their information processing so as to limit energy consumption while maintaining key behaviorally-relevant information. In this report, we re-examine classically-defined brain areas in the visual processing hierarchy, and ask whether neurons in these areas vary lawfully in how they represent sensory information. Our results suggest that neurons in these brain areas shift from being an optimal conduit for conveying sensory information towards prioritizing information that supports key perceptual discriminations during natural tasks.

## Introduction

An appealing theory of neural computation is that neurons in early sensory areas respond to stimuli in a way that maximizes the information carried about the world [1, 2] while neurons in downstream areas transform this representation to best support specific tasks and computations [3–7]. A careful test of this theory of sensory transformations requires several elements. We need to characterize the typical probability distribution of a behaviorally-relevant sensory variable encountered in the environment. We then need large-scale measurements of neural responses driven by this sensory variable across multiple brain areas. Lastly, we need a single mathematical framework that can be applied to sensory representations shaped by different behavioral or computational objectives. In this report, we use binocular disparity in the primate visual system as a model and combine the necessary statistical, physiological, and computational resources to test this theory. Our results provide empirical evidence in support of a systematic transformation of sensory representations in the brain: from information preservation at early processing stages to supporting perceptual discrimination performance at later stages.

**Fig 1:**
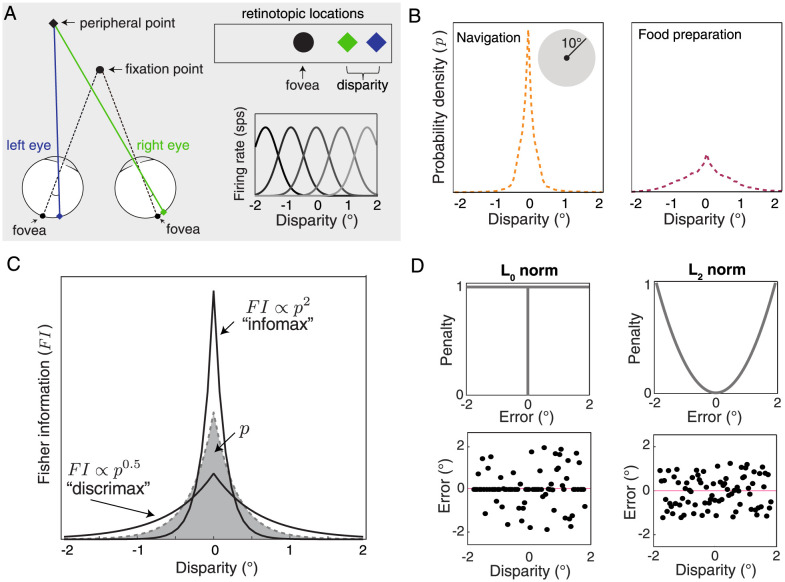
Statistical regularities in binocular disparity can be used to examine how sensory representations transform across visual brain areas. **A**. Non-fixated points tend to fall on disparate retinotopic locations because the eyes are laterally offset. The horizontal difference in these locations is called the horizontal binocular disparity (abbreviated here as simply disparity). Populations of neurons tuned for different disparities, illustrated in the bottom right, are found throughout the visual system. **B**. Disparities encountered in the central visual field (10° radius of fixation) tend to be small. Each plot shows the probability density of disparity obtained from data collected in [13] while human participants either navigated an outdoor environment (left) or prepared a sandwich (right). Densities are estimated from over 100 million samples of disparity recorded while three human participants performed these tasks. **C**. Information theoretic frameworks indicate that optimal sensory representations can be described by a power law relationship between stimulus probability *p* (shaded region) and the Fisher Information (*FI*) of a neuronal population. Information maximizing population codes (“infomax”) are proportionate to the squared probability, while discrimination maximizing codes (“discrimax”) are characterized by a compressive nonlinearity. **D**. The plots on the top illustrate normalized error functions that minimize the *L*_0_ (left) and *L*_2_ (right) norms. The plots on the bottom illustrate example patterns of disparity estimate errors under the respective norms used to optimize the reconstruction of the ground truth from noisy sensory measurements. For clarity, disparities are sampled from a uniform distribution. The sets of reconstruction error under the *L*_0_ and *L*_2_ norms have equal total error penalty. Red lines indicate zero error.

Binocular disparity between the retinal images offers an ideal test bed for examining hierarchical sensory representations. In animals with forward-facing eyes, non-fixated points in space tend to fall on disparate retinotopic locations because the eyes are laterally offset from each other (Fig 1A). Successful integration of information from the two eyes relies on populations of neurons that are tuned for different binocular disparities–the differences in the retinotopic location of points imaged in the left and right eyes. While neuronal tuning for binocular disparity emerges early in the mammalian visual system (V1), populations of neurons tuned for binocular disparity have also been characterized all along the dorsal and ventral processing streams [8, 9]. Beyond just supporting binocular integration, the sensing of horizontal binocular disparities in particular provides one of the most reliable cues to the relative distances of objects in the environment. As such, this cue supports a variety of perceptual tasks such as figure/ground segregation, three-dimensional motion perception, and breaking camouflage [10–12]. The magnitude and direction of horizontal binocular disparity varies lawfully as a function of how far objects are from the observer as well as where the observer is fixating, and prior work has shown that these variations result in predictable statistical regularities in the binocular disparities encountered during natural tasks [13–16]. We hypothesized that early representations of horizontal binocular disparity maximize the information carried about typical disparities encountered during natural behavior, while later representations may reallocate neuronal resources to facilitate discrimination of disparity in support of perceptual tasks.

## Results

### Natural distributions of binocular disparity have strong statistical regularities

To test this sensory transformation hypothesis, we first need an understanding of the distribution of horizontal binocular disparities that the visual system is tasked with processing (hereafter simply referred to as disparities). In recent years, there has been a concerted effort to characterize the visual “diet” of disparities that is typical of natural experience [13–16]. This prior work suggests several robust statistical properties of typical disparities, most notably that small disparities (near zero) tend to be much more likely than large disparities in central vision. This pattern likely arises because objects and surfaces tend to be locally smooth. When the eyes fixate on an object, the points nearby are mostly at a similar distance from the observer and therefore produce modest magnitudes of disparity. Optimal sensory representations for binocular integration should therefore allocate processing resources differently for small disparities than for large ones. Using a previously-collected data set in which people wore a custom-built gaze and scene tracker [13], we calculated the probability distribution of binocular disparities in the central visual field while people performed two different tasks: food preparation and navigation (Fig 1B). While the distribution shape differed between the tasks, we see that both distributions are approximately zero-mean, symmetric, and highly kurtotic (consistent with previous analyses). The increased prevalence of larger disparities during food preparation is likely due to the different typical object distances between the tasks: a given depth interval between two objects maps to a larger disparity if the objects are relatively close to the observer, as during manual tasks like preparing food.

### Sensory coding theories predict a lawful transition in how these binocular disparity statistics are reflected in the brain

Optimal coding frameworks provide concrete, testable predictions for how a stimulus probability distribution should be reflected in neural populations. Specifically, if a population maximizes the information carried about a sensory variable, we expect the Fisher Information of the population neural activity (*FI*; a measure of the precision with which a sensory variable is encoded) to follow a power law in which *FI* is proportionate to the stimulus probability squared (Fig 1C, “infomax”) [17]. Importantly, this relationship requires us to assume that *FI* is a reasonable proxy for another information theoretic quantity: the mutual information between the stimulus and the neuronal representation. Prior work suggests that this assumption holds best when the population-level noise is weak and Gaussian [18]. More generally, this representation can also be interpreted as a reference prior [19], which assumes the least possible information about the world, or Jeffrey’s prior, providing invariance across transformation of sensory units [20].

Under common assumptions on computational limits, this *FI* ∝ *p*^2^ power law corresponds to a neural code that minimizes the *L*_0_ norm of the stimulus reconstruction error [5, 6, 17, 18]. This means that all error magnitudes are penalized equally and the expected value of these errors is minimized (i.e., error sparsity is maximized; Fig 1D, left) [5, 6]. Prior work has found signatures of this power law across a range of early sensory brain areas [3, 21]. However, a representation with this distribution is not optimal for visual tasks that require discrimination between different values of a stimulus variable, because error magnitude matters for many discrimination tasks. For example, if one is trying to determine whether their hand can fit in between two sharp objects separated in depth, a small error may be harmless but a large error may lead to injury. Thus, for perceptual discrimination, neural codes that minimize some other error metric like mean squared error are often appropriate (*L*_2_ norm; Fig 1D, right). As such, codes that are optimized for downstream discrimination tasks should reduce the concentration of neural processing resources on high probability events and spread *FI* more equally across the stimulus space (Fig 1C, “discrimax”) [5, 18]. The discrimination-maximizing “discrimax” line shown in the Fig 1C corresponds to a specific power law of *FI* ∝ *p*^0.5^. This objective (along with objectives that aim to minimize other *L*_*p*_ norm errors like the sum of absolute errors) results in a consistently more compressive nonlinearity than information maximization [6, 22]. In neural codes for binocular disparity, we would therefore hypothesize that the population *FI* might be more strongly peaked at zero disparity in early visual areas, and more equally spread out across a broader range of disparities in later visual areas.

### Neural populations differ as predicted by a transition from information-preservation towards supporting perceptual discrimination

**Fig 2:**
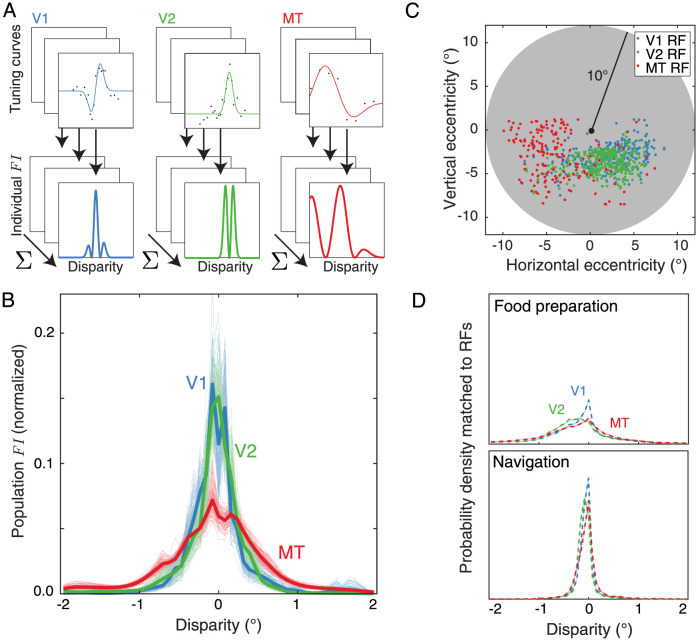
We calculated the population Fisher Information (*FI*) from sets of neurons from areas V1, V2, and MT and the probability density of disparities sampled to match their receptive field (RF) locations. **A**. We compiled a data set of 1007 disparity tuning curves from brain areas V1 (n = 388), V2 (n = 441), and MT (n = 178) of the macaque monkey. The mean response of each neuron as a function of disparity was fit with a continuous function and the *FI* associated with each tuning curve was calculated from these fits, assuming Poisson spiking. **B**. The population *FI* is shown for each brain area, assuming that each neuron responds independently (thick lines). Each curve is normalized to have the same sum, to facilitate comparing the shapes despite differences in the number of neurons per population. Thin lines represent the population *FI* computed from 100 bootstrapped samples from each brain area. **C**. Small circles show the locations of the centers of all receptive fields (RFs) of the neurons analyzed in each brain area as a function of horizontal and vertical eccentricity from fixation. The samples were used to compute kernel-smoothed densities as a function of visual field location. The shaded gray region indicates a 10° radius around fixation. **D**. On the top, we show the disparity probability densities collected from the food preparation task weighted based on the kernel-smoothed spatial sampling of the V1, V2, and MT data RFs. On the bottom, we show the same for the navigation task. The 95% confidence intervals of the disparity probability distributions across 100 bootstrapped samples are shown with shading, but the range is smaller than the thickness of the lines.

To test this specific hypothesis, we must characterize a large number of neuronal tuning curves for binocular disparity across different brain areas, such that we can calculate the population *FI* associated with these curves and compare them to the disparity probability distributions in Fig 1B. Since the precise shape of the disparity probability distribution varies between tasks (and different resource constraints can change the numerical value of the optimal exponent for the power law [5, 22]), here we focus on the *relative* transformation of the *FI* exponent between brain areas rather than on its nominal value. To this end, we compiled a data set of 1007 neurons’ binocular disparity responses spanning brain areas V1, V2, and MT of the macaque monkey, which were collected in the course of previously published studies [23–34]. The mean responses of each neuron as a function of binocular disparity were fit with a continuous 1D Gabor function and the *FI* associated with each individual neuron’s tuning curve was calculated from these fits based on the assumption of Poisson spiking (Fig 2A).

For a single neuron, the *FI* is high when the tuning curve is steep and the spike rate (and Poisson noise) is low, and the *FI* is low when the tuning curve is flat and the spike rate is high (for example, note the alignment between the tuning curve flanks and the *FI* peaks in Fig 2A). The total *FI* of each population was calculated as a sum across neurons, based on the initial assumption that each neuron responds to stimuli independently (Fig 2B). Qualitatively, we see that the population *FI* is most kurtotic in V1 and V2, and least kurtotic in MT, consistent with the hypothesis that the information-maximizing model is a better description of the early visual representation (V1 and V2) and the discrimination-maximizing model is a better description of the downstream representation (MT).

**Fig 3:**
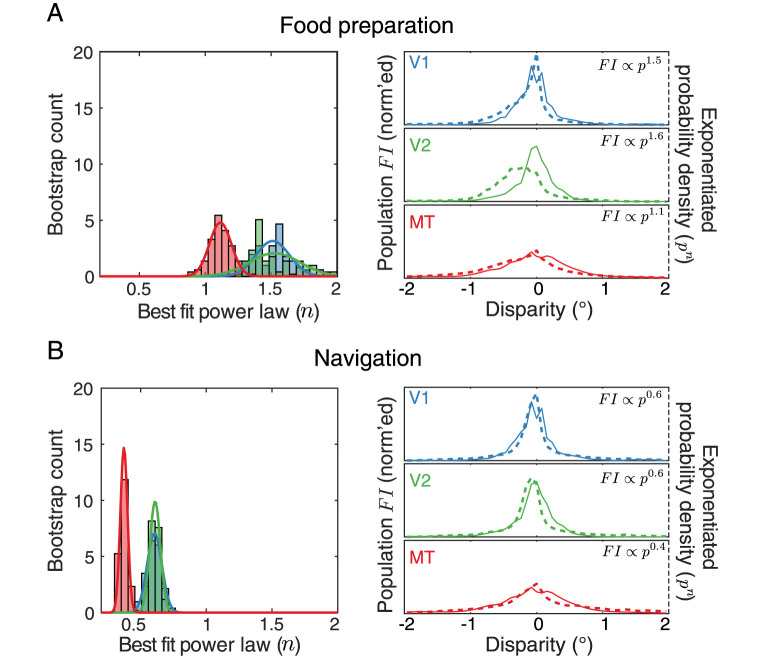
Neural populations differ as predicted by a transition from information-preservation towards supporting perceptual discrimination. **A**. On the left, the distribution of best-fit power law exponents linking population *FI* to the disparity probability densities is plotted for each brain area for the food preparation data set. Histogram bars indicate the 100 bootstrapped samples for each brain area and solid lines indicate Gaussian distribution fits to each set of samples. On the right, the normalized population *FI* is plotted for each area (solid lines) along with the disparity distributions scaled by their respective best-fit power laws (dashed lines). **B**. As in panel A, but for the navigation task.

We next directly compared these empirical *FI* distributions to the two probability distributions of disparities in the natural environment during food preparation and navigation. Before doing so, we had to recompute the disparity distributions based on the retinotopic locations of the neuronal receptive fields (Fig 2C). We did this because even in the central visual field, disparities are distributed somewhat non-uniformly across space. For example, negative (or near) disparities are more common in the lower visual field than the upper visual field [13]. Since the receptive field locations of our sample populations tended to fall in the lower visual field, we resampled the natural disparity distributions based on the specific retinotopic locations of the neuronal receptive fields in each population using kernel-smoothed density estimates (Fig 2D). We then calculated the power law that, when applied to the corresponding disparity distributions, resulted in the best fit to the measured *FI* of each neuronal population. That is, we found the value *n* that most closely satisfied the relationship *FI* ∝ *p*^*n*^ for V1, V2, and MT. We used bootstrapping of the populations to estimate variability of the best-fit power law exponent. Consistent with our hypothesis, we observed a systematic decrease in the best-fit exponents from the lower-level areas to the higher-level area for both natural tasks (food preparation: Fig 3A left panel; navigation Fig 3B left panel). Note that in both plots, the distributions for V1 and V2 are largely overlapping. The results of these fits suggest that V1 and V2 are closer to populations optimized to preserve information about binocular disparity (particularly for the food preparation task, for which the best fit exponents are 1.5 and 1.6, respectively). The best fit exponent for MT was lower for both tasks, and therefore less consistent with information maximization and more consistent with reducing error magnitude. Of course, the differences between V1/V2 and MT are not as extreme as the infomax and discrimax examples given in Fig 1, but the relative shift is robust and persistent across both disparity distributions.

However, we observed one notable inconsistency with this interpretation, with respect to V2 and the food preparation task. The right panels in Fig 3A and 3B show the matches between the population *FI* (solid lines) and the disparity distributions scaled by the single best fit exponents (dashed lines). According to our predictions, for each brain area and each task, these pairs of distributions should closely overlap. The distributions match closely in all cases except for the V2 population and the food preparation task. The exponentiated disparity distribution in this case is quite biased towards near (negative) disparities, and this bias is not present in the population *FI*. The reason for the near disparity bias comes down to the distribution of receptive field locations: the V2 receptive fields are exclusively concentrated in the lower visual field, and the disparity statistics in the lower visual field are strongly biased towards near disparities during food preparation [13]. For the navigation task, the binocular disparity statistics are less biased. At present, we do not understand how the visual system might flexibly incorporate different biases in stimulus statistics when they differ across tasks. However, we speculate that the notable lower visual field near bias in this food preparation task (in which human participants made a sandwich while sitting at a table) may be a bit weaker in the visual experience of macaque monkeys.

To quantify the exponent differences further, we fit the bootstrapped power law values for each neuronal population with a Gaussian distribution and measured the effect sizes between populations (Table 1, top row). The effect sizes, measured as Cohen’s D between pairs of populations, were large between the earlier areas and MT. As expected from the similarity in their *FI* distributions, the effect sizes were small between V1 and V2. Despite the differences in overall magnitude across the two tasks, the ratio of the best-fit exponents for the early (V1 and V2) and later (MT) brain areas were similar (Table 1, bottom row). Thus, the data support the notion that the MT population *FI* reflects a different optimization, but that V1 and V2 may contain similar information-driven codes.

**Table 1:** Comparison of best-fit power law exponents between brain areas. Pairs of areas being compared are indicated at the top of each column. For each task, the upper values indicate the effect sizes (Cohen’s D) between the bootstrapped exponent distributions. The lower values indicate the ratios of the single best-fit exponents.

statistic	V1 vs. V2		V1 vs. MT		V2 vs. MT	
	food	nav	food	nav	food	nav
effect size	0.1	0.1	3.7	5.3	2.8	6.9
exp. ratio	1.0	1.0	1.3	1.7	1.4	1.7

### These differences are robust to assumptions about neuronal noise and information-limiting correlations

**Fig 4:**
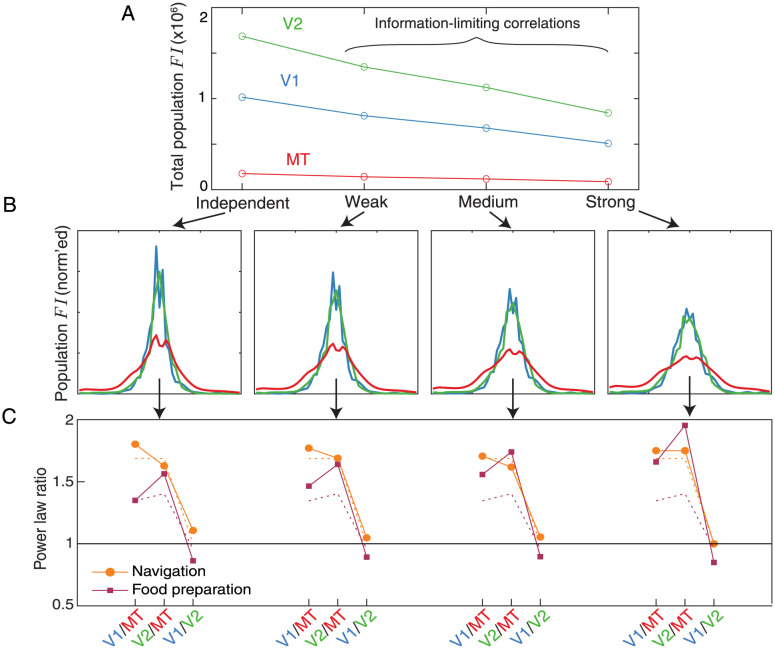
Differences in the best-fit power law across areas are robust to assumptions about neuronal noise and information-limiting correlations. **A**. We introduced four control models to explore how our model of neuronal noise might affect the conclusions drawn from our main analysis. For each control model, we plot the total *FI* (the area under the curve of the population *FI* distribution). The independent model is computed much like the original Poisson spiking model, but we fit a per-neuron noise model, summarized in Table 2. The weak, medium, and strong models further contain information-limiting correlations that reduce the total population *FI* in each area by a factor of 1/5, 1/3, and 1/2, respectively. Note that the differences between areas also reflect the different number of neurons in each population that we analyzed. **B**. For each of these models, the shape of the normalized population *FI* (in which each distribution sums to one) is plotted. The x and y axes, which are omitted for clarity, are identical to Fig 2B. **C**. For each model, we plot the ratios between all pairs of best-fit power law exponents for both tasks. For reference, the ratios associated with the best-fit power laws determined from the independent Poisson noise model are shown as dashed lines.

The preceding analysis assumes that each neuron in V1, V2, and MT fires with independent Poisson variability—that is, that the variance of the spike count is equal to the mean spike count for each stimulus disparity, measured over a given time interval. However, systematic violations of Poission spiking statistics have been reported in a number of neuronal populations [35–37]. If, for example, spike count statistics varied systematically between V1, V2, and MT in our dataset, our analysis of the differences in population *FI* may be flawed. In a control analysis, we thus fit the mean and variance of the spike counts for each neuron in our dataset and used these fits to determine the variance at each disparity in our analysis (assuming Gaussian-distributed noise). Because the number of repeated measurements was limited, these fits were quite variable (Table 2), but were consistent with the observations from prior work that deviations from Poisson spiking tend to be associated with slopes greater than one and intercepts greater than zero [37]. In addition to considering unique individual neuron variability, it is also highly likely that these populations contain noise correlations that are not reflected in the current, non-simultaneous, recordings. As such, we also augmented our model to examine the potential impact of noise correlations. We focused on a particular type of pairwise correlation called “information limiting correlations” [38], since these correlations will have the most notable effect on the *FI* contained in large neuronal populations. We modelled three levels of information-limiting correlations, which collectively reduced the total population *FI* in each population by a factor of 1/5, 1/3, and 1/2 (Fig 4A).

**Table 2:** A constrained least squares algorithm was used to fit a slope and intercept to the relationship between mean spike rate and spike rate variance for each neuron in each area. Column values indicate the median fits, with the 25th and 75th percentiles in parentheses. The intercepts were constrained to be greater than zero (hence the lower quantile being approximately zero for each area).

area	slope	intercept
V1	1.4 (0.8,2.1)	0.6 (∼0,16.6)
V2	1.4 (0.8,2.2)	1.4 (∼0,7.8)
MT	1.3 (0.3,2.4)	15.5 (∼0,67.9)

Fig 4B shows the resulting shapes of the population *FI*, for each of these four additional noise models. The per-neuron fitted independent noise model (leftmost plot) produces *FI* distribution shapes quite similar to the Poisson noise model. Interestingly, as information-limiting correlations were increased, we found that the shape of the population *FI* in each area tended to broaden. These correlations seemed to have a disproportionate effect on the population *FI* at small disparities, where each population was most informative. However, since this broadening affected all brain areas, we hypothesized that the ratios between the best-fit powers may be largely preserved. In Fig 4C, we plot these ratios for each noise model, along with the ratios associated with the independent Poisson model for comparison (dashed lines). In all models, the ratio between the best-fit powers of V1 and MT, and V2 and MT, tends to fall in the range of 1.4-2. The ratio between V1 and V2 stays near 1. Thus, this analysis suggests that the proposed information theoretic differences between these brain areas are likely robust to the inclusion of information-limiting correlations that are similar between areas. If there were strong differences in the correlation structure between brain areas, however, that could reduce the differences in coding precision shown here.

### These differences correspond to a broad set of changes in individual tuning curve characteristics from V1/V2 to MT

**Fig 5:**
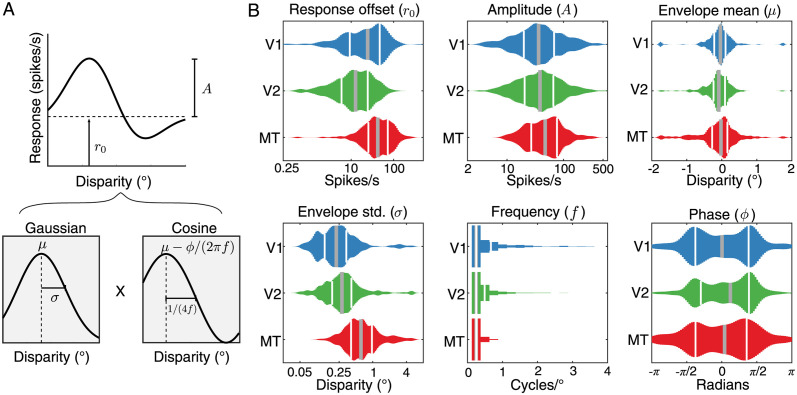
Tuning curve characteristics differ systematically across areas. **A**. Decomposing the 1-D Gabor function that was fitted to each neuron’s mean responses into the Gaussian (left plot) and cosine (right plot) components clarifies what each parameter contributes to the shape of the resulting tuning curve. The vertical dashed line in the Gaussian plot marks the center of the Gaussian envelope while the cosine plot indicates the 0 phase position. The parameters are defined as follows: *A*: Amplitude, *r*_0_: vertical response offset, *μ*: Gaussian envelope mean, *σ*: Gaussian envelope standard deviation, *f*: cosine frequency, *ϕ*: cosine phase. The cosine *f* parameter is shown in the bottom right panel as defining one quarter of a period. **B**. Distributions of best fitting Gabor parameters for each of the three areas. Thin white bars indicate the 25th and 75th quartile and the thick gray bar indicates the median.

We next asked which aspects of the neural responses to disparity could account for the differences in the *FI* distributions between V1/V2 and MT. To answer this question, we leveraged the Gabor fits to each of the tuning curves (Fig 5A). For each of the cortical populations, we examined the distributions of each of the six best-fit Gabor parameters (Fig 5B). Omnibus Kruskal-Wallis tests (used because some parameters were not normally distributed) indicated group-level differences in all of the parameters (Table 3). Follow up pair-wise tests indicated that the MT neurons generally had higher response offsets, higher response amplitudes, larger (absolute) envelope means, broader envelopes, and lower disparity frequencies than either V1 or V2 (Table 4). This analysis expands on a previous comparison between V1 and MT [34] by including responses from V2 and a larger number of V1 neurons. One possible explanation for these differences is that they may reflect differences in the retinoptic locations of the receptive fields across the samples from each brain areas, rather than systematic differences between these brain areas. Our subsampling from the initial larger data set resulted a good match between the brain areas in terms of eccentricity and vertical position within the visual field, although the MT data set is more concentrated in the left visual field and the V1/V2 data sets are more concentrated in the right visual field (see Fig 2C). Since there is no reason to hypothesize that disparity tuning should differ in terms of left or right visual field, we propose that these tuning differences most likely reflect differences in the underlying neural representation across the populations.

**Table 3:** Test statistics and significance for Kruskal-Wallis one-way test for differences between distribution medians comparing the six Gabor parameters across V1, V2, and MT (distributions shown in Fig 5B). For the envelope mean and the cosine phase, statistics were run on the absolute value. The degrees of freedom for each test is 2. Bolding indicates a statistically significant difference between the brain areas, with a significance threshold of *p* < 0.05.

parameter	*χ* ^2^	p-value
*r*_0_	187.8	**<0.001**
*A*	10.0	**0.007**
|*μ*|	16.3	**<0.001**
*σ*	208.5	**<0.001**
*f*	52.5	**<0.001**
|*ϕ*|	7.0	**0.030**

**Table 4:** Test statistics and significance for two-tailed Wilcoxon rank sum test for differences between medians, used for pair-wise follow up tests on the Kruskal-Wallis tests from Table 3. Pairs of distributions tested are indicated at the top of each column. Test statistics (*z*), effect sizes (*r*), and p-values (*p*) are provided for each pair. For the envelope mean and the cosine phase, statistics were run on the absolute value. Bolding indicates a statistically significant differences between the brain areas, with a significance threshold of *p* < 0.05.

parameter	V1 vs. V2			V1 vs. MT			V2 vs. MT		
	*z*	*r*	*p*	*z*	*r*	*p*	*z*	*r*	*p*
*r*_0_	8.0	0.28	**<0.001**	-6.9	0.29	**<0.001**	-13.3	0.53	**<0.001**
*A*	-0.2	0.01	0.845	-2.9	0.12	**0.004**	-2.9	0.12	**0.004**
|*μ*|	-1.3	0.05	0.193	-3.9	0.17	**<0.001**	-3.2	0.13	**0.002**
*σ*	-4.9	0.17	**<0.001**	-13.6	0.57	**<0.001**	-11.8	0.47	**<0.001**
*f*	2.8	0.10	**0.005**	6.8	0.29	**<0.001**	5.7	0.23	**<0.001**
|*ϕ*|	2.5	0.09	**0.013**	-0.02	0.00	0.986	-1.8	0.07	0.076

### An increase in neurons preferring larger disparities in MT is a key factor in the observed sensory coding transformation

**Fig 6:**
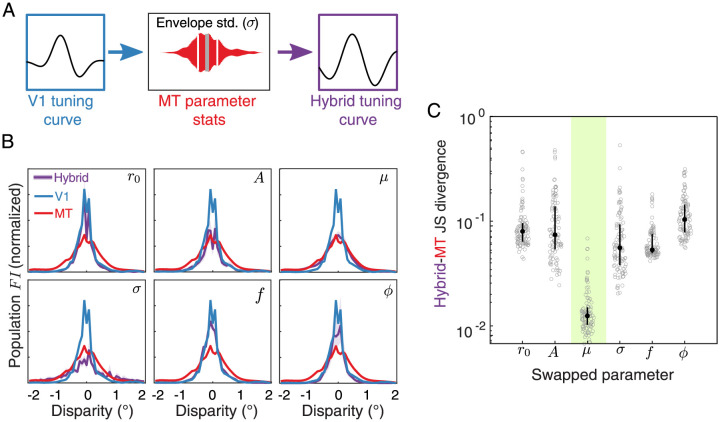
An increase in neurons preferring larger disparities in MT is a key factor in the observed sensory coding transformation. **A**. We investigated which of the six Gabor parameters best explained the difference in the population *FI* between V1 and MT by replacing one parameter at a time from V1 with randomly sampled values pulled from the distribution of best-fitting parameters from the MT population. We repeated this process to create 100 new hybrid populations for each of the 6 parameters. **B**. The median and interquartile range of the population *FI* distributions from the 6 hybrid populations (purple) are plotted against the true V1 (blue) and MT (red) population *FI* (same in each of the 6 subplots). **C**. Jensen-Shannon (JS) divergence between the hybrid population *FI* distributions and the true MT population *FI* distribution are plotted for each swapped parameter. Lower values reflect a closer match between the two distributions. Gray circles show the JS divergence between the population *FI* for each of the 100 hybrid bootstrapped populations; black circles and errorbars show the median and interquartile range across all bootstraps.

There are clear differences in the distributions of best fit Gabor parameters between the earlier cortical areas and MT. However, the complex and overlapping effects that these parameters have on the tuning curve shape make it hard to interpret how each parameter contributes to the transformation of the population *FI* shape between the areas. Therefore, we performed a resampling analysis to see if the changes in any one parameter in particular had a notable impact on the difference in the *FI* distributions. Since the *FI* distributions from V1 and V2 were similar, we focused this analysis on comparing V1 and MT and used the independent Poisson noise model. The overall approach is outlined in Fig 6A: for one tuning curve parameter at a time (illustrated just for envelope standard deviation), we replaced the set of true V1 values with a new set of values obtained by randomly sampling from the distribution of MT fits. We then rebuilt each cell’s tuning curve with their new parameter and used these hybrid cells to calculate a new *FI* distribution. This process was repeated a total of 100 times for each of the Gabor parameters individually to assess the variability in the resulting hybrid population *FI* distributions. Fig 6B shows the hybrid *FI* distributions (purple) for each of the parameters alongside the original V1 and MT population *FI* distributions (blue and red, respectively). Of the six parameters, replacing the V1 envelope mean (*μ*) with those from MT qualitatively results in the closest match with the true MT population.

To examine these matches further, we calculated the Jensen-Shannon (JS) divergence between the *FI* of each of the hybrid populations and the true MT population (Fig 6C). This information theoretic measure reflects the dissimilarity between two distributions, such that a low value indicates that two distribution are similar and a high value indicates that two distributions are dissimilar. We observed that the median JS divergence associated with resampling the envelope mean (*μ*) was a factor of 4.0-7.6 lower than the medians for the other parameter resamplings—that is, it was the most similar to the true MT distribution (green shading). We performed two control analyses to examine the generality of these results, which are described in the Methods. A limitation of this approach is that it does not consider correlations between parameters. For example, a closer match between the MT and the hybrid populations may be achieved if we matched both the envelope mean and width. However, focusing on single parameters allows us to gain a simple intuition about how the known differences in MT disparity tuning affect the distribution of information therein. We conclude that an increase in neurons preferring larger disparities may be a key driver of the observed sensory information transformation from V1 and V2 to MT.

## Discussion

Here we have taken a theoretical prediction about changes in population-level neuronal information along the sensory processing hierarchy and put it to an empirical test. The results are consistent with the prediction that sensory transformations can be understood as the result of a constrained optimization, in which the goal changes sensibly from early to later sensory areas.

While we focused on how different encoding objectives might affect the power law relationship between stimulus probability and population *FI*, the optimal population *FI* can be influenced by other factors as well, such as resource constraints [22]. We assumed that visual brain areas in the same species are subject to the same constraints, which seems reasonable given the overall similar anatomy and physiology of these areas. However, adaptation studies that dynamically shift stimulus statistics without affecting constraints may be able to determine whether or not this assumption holds [39]. Similarly, system noise influences the form of *FI*, both for individual neurons and the population as a whole (see [40]). The noise analysis of our data suggests that, at least in the current data set, the results are robust to different simulated noise properties and noise correlations. However, a systematic characterization of neuronal noise properties and noise correlations along the sensory processing hierarchy may ultimately reveal that sensory transformations are affected by differing noise properties across brain areas as well. For example, effects of spatial attention on response variability have been shown to change from earlier to later stages of visual processing [41, 42]. Evidence suggests that attention-related response changes may also produce neuronal correlations that are amplified as signals move downstream, which can be measured as increasing deviations from Poisson spiking statistics [37]. While our analysis of the spiking properties of V1, V2, and MT neurons in response to disparities did not reveal systematic changes in the best-fit slope between each neuron’s spike rate mean and variance, we did see that the overall spike rate in MT was notably higher on average than in V1 and V2 (Table 2). Advances in our ability to model how different coordinated processes between neurons translate to spiking statistics and correlations will help us ultimately understand how processes like attention and arousal affect sensory information [43]. Similarly, we hope that the promising empirical results like those presented here will motivate further collection of simultaneous population-wide activity for examining how information changes within hierarchical sensory representations.

In this study, we used binocular integration as a model system for sensory transformations more generally. But our results also shed light on specific open questions regarding the neural underpinning of binocular integration and disparity processing. Previous work has noted that the shape of disparity tuning curves appears to change systematically across brain areas, but the reason for these changes is unknown [34, 44, 45]. For example, previous work suggested that MT tuning curves tend to have odd symmetry and broader tuning, whereas in V1, the tuning curves are more even symmetric with a narrower range of preferred disparities [34]. Here, we did not see evidence for a clear difference in even/odd symmetry, but we did observe a multifaceted set of differences in the distributions of tuning curve shapes. Some of the difference in shape (e.g., width) may be a side effect of pooling neurons with different orientation preferences to generate direction selectivity for patterns [46]. However, a priori, it is not necessarily the case that tuning curves get broader along the sensory processing hierarchy. For example, the tuning curve characteristics in V2 were quite similar to V1. Our information theoretic analysis suggests these differences in tuning curve shape may also have direct utility: they shift the position of a neuron’s peak *FI* to larger disparities while maintaining a population peak near zero. Over the entire population, this effectively makes the *FI* distribution more broad, thus preserving disparity discrimination at higher disparity pedestals while allocating (relatively) fewer resources to small disparities. Given that each area of the brain is subject to resource limitations and input-output noise, redistributing visual information so as to more closely match the aspects of visually-guided behavior supported by each area seems to make sense. It would be interesting, for example, to examine the *FI*-based sensory representation of disparity in a ventral stream area such as V4, where it has been proposed that disparity representations support detection of fine 3D structural elements [44].

Of note, we do not want to suggest that a shift from information-maximizing towards discrimination-maximizing representations should be the sole difference in how V1, V2, and MT represent visual information. For example, it is well-established that MT encodes binocular disparity in a way that is more correlated with perception than V1 does: V1 neurons invert their disparity tuning curves with anticorrelated stereoimages [8] and are affected by vergence and absolute disparity [47, 48], whereas V2 [49, 50] and MT [51] at least partially discard false stereo matches and encode relative disparity. It is also important to consider other sensory variables represented in these areas. A recent study used an information theoretic framework to examine the link between the encoding of speed in MT and perceptual biases in speed estimation [52]. They found that the population *FI* for stimulus speed in MT can be related to speed perception via an *FI* ∝ *p*^2^ power law. Our estimates of the power law in MT for binocular disparity diverge from this idealized “infomax” representation of speed. However, it is not necessary that every sensory variable encoded within a population is represented at the same level–it is entirely possible that the same brain area could contain an information-maximizing representation of one sensory variable and a discrimination-maximizing representation of another. The information-theoretic framework provides an additional window into how neural representations build and interact along sensory processing streams that can complement other assessments of neural function.

Lastly, our work also highlights the fact that the statistics of sensory input can be task dependent, because the disparity statistics were quite different for the two tasks that we considered (food preparation and navigation). This task-dependence can pose a problem for assessing the encoding optimality of neural populations on the basis of task-free natural stimulus statistics derived from generic datasets, for example, of natural images and sounds [3, 4]. Inter-species differences in sensory statistics also pose a challenge. The statistics associated with natural experiences and tasks may be easier to measure in some species than others, however, differences in the ecological niches and behaviors can dramatically affect the patterns of sensory experience [53]. Here, we show that generalizations that are robust across tasks, and potentially across species, can be made by focusing on relative differences between brain areas.

With the information theoretic analyses like those presented here, we gain a more principled understanding of the link between the hierarchy of cortical areas carrying sensory information and the complexity of behaviors that rely on that information. New computational frameworks that can be applied to dynamic populations of neurons, trial-by-trial variations, and spike-count correlations between neurons will contribute to the next steps in characterizing hierarchical transformations of sensory signals.

## Methods

### Definition of horizontal binocular disparity

We define horizontal binocular disparity (*d*) as follows:

$$\begin{array}{c} d=\beta_{L}-\beta_{R}, \end{array}$$ (1)

where *β*_*L*_ and *β*_*R*_ denote the horizontal angular eccentricity of an image projected to the left and right retinas, relative to the fovea. We represent eccentricity and binocular disparity in units of visual degrees, with negative disparity values indicating points that are closer in depth than the fixation point (crossed disparities) and positive values indicating points that are farther in depth than the fixation point (uncrossed disparities).

### Natural scene statistics of binocular disparity

Measurements of binocular disparity during natural tasks were re-analyzed from a previously collected dataset, in order to estimate the natural probability distributions of disparity (*p*(*d*), abbreviated as simply *p* in the main sections) [13]. In brief, three adult human subjects performed either outdoor navigation or food preparation tasks while wearing a custom-designed headset consisting of a pair of stereocameras and eye trackers. Still images were sampled from the stereocamera video footage, transformed into head-centered 3D scene geometry using an automated stereoscopic depth estimation algorithm, and finally converted into binocular disparity maps in retina-centered coordinates using the eye-tracking data. Analyses were limited to data within a 10° radius of the point of fixation and constituted 8670 disparity maps for the navigation task and 9551 disparity maps for the sandwich taking task (over 100 million disparity samples per task).

While the original natural disparity measurements were sampled within a 10° radius of fixation, the neural data sets we wanted to compare them to contained neurons with receptive fields that were restricted largely to one hemifield (Fig 2C). Given the differences in binocular disparity statistics between the upper and lower hemifields and the expected increase in prevalence of larger disparities with increasing eccentricity [13], these biases on receptive field location likely influence the probability distribution of disparities that each neuronal population encodes. Thus, we resampled disparities based on the retinotopic locations of the receptive field centers. To do so, we calculated kernel-smoothed probability density distributions for each of the cortical areas using a 2D Gaussian kernel and used these distributions to subsample from the original disparities in space. We then computed the overall probability density across 51 disparities linearly spaced between -2° and +2° (Fig 2D). To ensure the reliability of this subsampling procedure, we used bootstrapping to estimate the variability of the resulting distributions for each task. Over 100 iterations, we sampled a random set of 100 disparity maps with replacement and recomputed the disparity distributions. In practice, the bootstrapped 95% confidence intervals were smaller than the line width in Fig 2D.

### Neural recordings

Neural recordings from areas V1, V2, and MT were re-analyzed from multiple previous studies. All recordings come from awake fixating macaque monkeys and reflect measured action potentials (spikes) of isolated neurons. The tuning curves measured in V1 and V2 come from multiple studies using highly similar methods measured over the course of a decade in the same laboratory [23–33]. The MT tuning curves were obtained in a single study with slightly different experimental methods [34].

In all cases, the stimuli used to measure response rate (spikes per second) as a function of binocular disparity were random dot stereograms (RDS) with each dot subtending approximately 0.1°. The methods for the stimuli and data collection for the V1 and V2 data are described in [24, 27]. Briefly, responses from V1 and V2 were collected using RDS stimuli with no coherent motion, presented on a Wheatstone mirror haploscope for 400-500ms at a display refresh rates ranging from 72-100Hz. Responses from MT were collected using RDS stimuli with 100% coherent motion tailored to each cell’s preferred direction, speed, and size [34]. For each of the MT recording sessions, stereoscopic stimuli were presented for 1500ms on a single monitor with liquid crystal shutter glasses at a refresh rate of 50Hz for each eye (cross-talk was measured to be <3%). For comparability with the V1 and V2 data, here we analyze the MT responses just from the first 500 ms of each trial. The stimuli in the MT recording sessions were also presented against a non-overlapping background of stationary dots at zero disparity to anchor vergence (this vergence lock was located outside of the neuronal receptive fields in all cases).

Recordings from each area were likewise similar. In brief, after the receptive field of each neuron was localized, the stimulus was adjusted to the optimal size (and velocity for MT) and the responses to different magnitudes and directions of binocular disparity were recorded. For the MT recordings, the majority of tuning curves were mapped using stimuli with disparities that ranged from −1.6° to 1.6° in steps of 0.4°, however for a few neurons larger disparities were used. For the V1 and V2 recordings, the stimulus disparity ranges were variable across neurons, with steps ranging from 0.029° to 1.2°. Responses from MT and V2 neurons were collected solely with single electrodes, while responses from V1 were collected mostly from single electrodes and a few from multicontact probes [32]. The receptive fields of neurons in V1 and V2 were largely restricted to the lower visual field due to cortical topography and the positioning of the implanted recording cylinders. In the MT data set, most recording cylinders were placed above the right hemisphere, so the receptive fields are largely limited to the left visual field. Horizontal and vertical eccentricity of the stimuli and receptive field positions were calculated by taking the arctangent of the distance on the screen from the fixation point and the viewing distance.

There were slight differences in experimental protocols between the laboratories (i.e., shutter glasses vs. mirror haploscope, coherent motion vs. incoherent motion), but these differences are unlikely to cause the differences in the *FI* distributions estimated from the data. For example, Palanca and DeAngelis (2003) compared disparity tuning in MT for moving and stationary dots, and the disparity tuning curves were very similar (although responses were generally weaker for stationary dots) [54]. Indeed, motion and disparity are independently encoded in MT [55]. Since MT receives disparity and motion signals from V1 and V2, it is likely that disparity and motion are also coded independently in lower visual areas. As such, the difference in motion energy between the stimuli used by the two laboratories is unlikely to produce consistent biases in disparity tuning. Most neurons in V1 and MT also do not show a dependence of disparity selectivity on interocular delay, instead showing an inverse relationship between response gain and interocular delay; those that do show disparity-delay inseparability do not exhibit large tuning shifts over the display intervals used in either of the data sets [27, 56].

### Tuning curve analysis

A subset of the neural recordings described in the previous section were selected for analysis according to set of inclusion criteria. First, we only included neurons with an average of ≥ 3 repeats per stimulus disparity. We then selected neurons for which a significant amount of the trial-by-trial variance in responses was explained by the disparity of the stimulus (one-way ANOVA at a significance level of p < 0.01). For the V1 and V2 recordings, the range of stimulus disparities presented was variable, so to ensure sufficient data to obtain a reliable fit to the tuning curves we only analyzed neurons with a range of at least 1° between the nearest and the farthest disparity tested.

In each study, stimulus disparity was recorded in screen coordinates rather than retinal coordinates. For example, a point with the same horizontal coordinate on screen for both eyes was coded as having zero disparity. However, in retinal coordinates, the locus of points with zero disparity is a circle that contains the fixation point and the optical center of the two eyes. Therefore, a point with the same horizontal coordinates on a planar screen will have an uncrossed (far) retinal disparity. In order to match up the neural recordings to the retinal disparities measured in the scene statistics analysis, we therefore applied a correction factor. In brief, the retinal eccentricity of each on-screen stimulus in the left and right eye was determined based on the screen distance, the horizontal screen coordinates in the left and right eye, and an assumed interocular separation of 30mm. These retinal eccentricities were used to calculate the angular disparity on the retinas, which was used in the subsequent analyses. Since the calculation of retinal disparity is sensitive to eccentric fixation points, we limited our analysis to neurons in which the responses were measured while the animal was fixating straight ahead.

For each of these neurons, we then fit a continuous tuning curve to the mean responses as a function of the stimulus retinal disparity *h*(*d*). Tuning curves were parameterized as a 6-parameter Gabor function (Fig 5A):

$$\begin{array}{c} h\left(d\right)=r_{0}+A\;\text{exp}\;[\frac{{\left(d-\mu \right)}^{2}}{2\sigma^{2}}]\;\text{cos}\left(2\pi f\left(d-\mu \right)+\phi \right). \end{array}$$ (2)

Best-fitting parameters for each neuron were determined using constrained non-linear optimization in MATLAB (Mathworks, Inc.), minimizing the mean squared error (fmincon). To prevent fits that deviated substantially from the observed range of spike rates, data were up-sampled by a factor of 2 using linear interpolation prior to fitting. Bounds for parameters were as follows: 0 < *r*_0_ < 500, 0 < *A* < 500, −1.75 < *μ* < 1.75, 0 < *σ* < 5, 0 < *f* < 4.5, −2*π* < *ϕ* < 2*π*. Fits that resulted in a minimum spike rate of less than 0.05 spikes per second or with a frequency (*f*) of less than 0.25 were strongly penalized (by multiplying the current error by 10^7^), to avoid instability in the calculation of Fisher Information and the interpretation of the fitted parameters, respectively. The optimization was initialized at 200 randomly selected starting points and optimized according to an interior point algorithm. The parameters with the lowest error across all initializations were taken as the final fit. A subset of neurons (less than 30, mostly in MT) were identified with poor fits on manual inspection, so fitting routines were re-run for these neurons with minor adjustments to the parameter ranges.

We then further subsampled the neuronal populations to ensure that each neuron included in our analysis was well-fit with a Gabor tuning function (we select only neurons with *R*^2^ ≥ 0.75), had RF centers within the defined region of the disparity image set (i.e., ≤ 10° eccentricity), and had RF centers within the same general subregion of the visual field. To enforce the final criterion, we determined the largest vertical and horizontal components of the RF centers from the V1/V2 data sets to define a rectangular bounding box and then selected only MT cells with RF centers within this region. This was enforced because the full MT data set contained a larger number of cells with RF centers in the upper visual field, which could potentially bias the disparity preferences of the sample [13]. The final cell counts from each sample were 388, 441, and 178 from V1, V2, and MT, respectively.

To investigate differences between the resulting parameter distributions (Fig 5B), we first performed a Kruskal-Wallis omnibus test to ask if there were any significant differences in median values between the three areas. Since we were primarily interested in differences in distribution breadth, we first took the absolute value of the signed parameters (*μ* and *ϕ*) to test only difference in the median magnitudes of these values. We then followed up with a set of Wilcoxon rank sum tests between each of the pairs of areas. Following the method described in [57], we computed the effect size (*r*) for each rank sum test as the ratio of the *z* test statistic to the square root of the total number of samples. The results of these tests are presented in Tables 3 and 4.

### Fisher Information

The Fisher Information as a function of disparity for an individual neuron (*y*) with tuning curve *h*(*d*) can be written as:

$$\begin{array}{c} FI_{y}\left(d\right)=\frac{h^{\prime 2}\left(d\right)}{\sigma_{h}^{2}\left(d\right)}, \end{array}$$ (3)

where *h*′(*d*) denotes the first derivative of the tuning curve and $\sigma_{h}^{2}\left(d\right)$ denotes a function describing the variance of the neuron’s spike rate as a function of the stimulus disparity.

Based on the simplifying assumption that the spiking for each neuron measured within a given time window is Poisson-distributed, in our main analysis we calculated the Fisher Information of neuron *y* (*FI*_*Py*_(*d*)) as described by [17, 58]:

$$\begin{array}{c} FI_{Py}\left(d\right)=\frac{h^{\prime 2}\left(d\right)}{h(d)}. \end{array}$$ (4)

Further assuming that each neuron’s spike rate is also conditionally independent, the Fisher Information associated with the whole population (*FI*_*P*_(*d*)) is:

$$\begin{array}{c} FI_{P}\left(d\right)=\sum_{y}FI_{Py}\left(d\right). \end{array}$$ (5)

This quantity, along with all other population-level Fisher Information quantities, is referred to as just *FI* in the main sections.

Thus, we evaluated the tuning curve of each neuron over the same 51 disparities used to compute the natural disparity distributions, computed *FI*_*Py*_(*d*) (Fig 2A), and summed together all neurons in a given area (Fig 2B). The Gabor fits occasionally resulted in small negative tuning curve values, so to facilitate stable calculations we replaced these with very small positive numbers. Specifically, any tuning curve values below the 5th percentile across all spike rates within the population were set equal to that percentile prior to summing the population (replacing with zero would result in infinite information). To compare the distribution shape across areas, we normalized each population distribution to sum to one.

To examine the robustness of the estimated population *FI*_*P*_(*d*), we repeated this analysis by bootstrapping samples of 200 neurons (sampled with replacement) 100 times for each area. The sample size of 200 was selected so that we could approximately match sample sizes across all three populations.

### Alternative noise models

To examine how closely the neurons in our samples adhered to the assumption of Poisson spiking, we also fit the individual variance/mean relationship for each neuron. For each neuron, we first computed the response mean and variance for all stimuli that had at least 3 repeats, assuming that the response distribution was approximately Gaussian. We then used the constrained least squares solver in Matlab (lsqlin) to find the best-fit slope (*a*) and intercept (*b*) for estimating the response variance as a function of the response mean:

$$\begin{array}{c} \sigma_{h}^{2}\left(d\right)=a\left(h\left(d\right)\right)+b, \end{array}$$ (6)

subject to the constraint that *b* was greater than zero (i.e., the variance for a zero mean response could not be negative). The distribution of fit parameters are reported in Table 2 for each brain area. Using this equation, we could then recompute the population Fisher Information using the more general form (Eq 3) and summing over the population (Fig 4B, left-most plot). Small values in the denominator were replaced as described in the previous section.

To incorporate pairwise noise correlations (i.e., non-independence), we followed the approach described in Moreno-Bote et al. (2014) [38]. First, we can generalize the calculation of population Fisher Information at each disparity as follows:

$$\begin{array}{c} FI_{C}\left(d\right)={h^{\prime }\left(\text{d}\right)}^{T}\Sigma {\left(d\right)}^{-1}h^{\prime }\left(\text{d}\right). \end{array}$$ (7)

Here, the bolded **h**′(d) indicates a vector of the first derivatives of all the tuning curves at disparity *d*, Σ(*d*) is a covariance matrix, and the subscript *C* indicates that the Fisher Information includes covariance. This is a simplified approximation of the full Fisher Information for correlated neuronal populations, which, for example, assumes Gaussian variability and omits a second trace term; this approach has been previously described in more detail and adopted in [38, 59–62].

Correlations in neural populations can take many forms. For example, neurons that are in close spatial proximity or that have similar tuning properties tend to be more highly correlated [63, 64]. However, these “limited-range” correlations generally do not limit population information for diverse populations [38, 65], and we confirmed this in simulations using our neural populations (not shown). Thus, we focused on simulating so-called information-limiting correlations [38], in which the covariance terms for each disparity are determined by the products of the tuning curve derivatives:

$$\begin{array}{c} \Sigma \left(d\right)=\Sigma_{0}\left(d\right)+\alpha h^{\prime }\left(\text{d}\right){h^{\prime }\left(\text{d}\right)}^{T}. \end{array}$$ (8)

In this equation, *α* indicates a scale factor determining the strength of the covariances, and Σ_0_(*d*) denotes a base covariance matrix that these information-limiting relationships are applied to. In practice, we first calculated the off-diagonal information-limiting covariances and then replaced the matrix diagonal with the original response variance of each neuron to avoid modifying the original variances. We performed a grid search to determine the *α* values for each area that caused a reduction of 1/5, 1/3, and 1/2 in the total population Fisher Information, as measured via the area under the curve (Fig 4A) and examined the effects on the shape of the normalized Fisher Information (Fig 4B). That is, each brain area had a customized set of scale factors. We converted the resulting covariance matrices to correlation values and confirmed that even the strong information-limiting relationships did not tend to create atypically-high pairwise correlations (e.g., most of the correlations in the strong condition fell within a range of -0.2 to 0.2).

### Comparison between Fisher Information and disparity statistics

We used a grid search to determine the power law exponent that minimized the difference between each population *FI* and the sampled binocular disparity probability distributions. Power laws were applied to the disparity distributions and then differences were calculated as the mean absolute error between the two distributions sampled at 51 evenly spaced binocular disparities between −2° and +2°. To summarize the effect size between brain areas, for each of the noise models we computed the ratios of the best-fit power laws between V1 and MT, V2 and MT, and V1 and V2 (Fig 4C). For the independent Poission model, we also repeated this minimization for each of the 100 bootstrapped samples and fit the resulting distributions with a Gaussian distribution using maximum likelihood estimation, which allowed us to characterize the effect size in terms of Cohen’s D between each pair of brain areas (Fig 3).

### Gabor parameter resampling between cortical areas

To identify which aspect of disparity tuning could best explain the differences in the *FI* distributions between populations, we conducted a resampling approach in which we replaced the best-fitting Gabor parameters individually for each parameter (*r*_0_, *A*, *μ*, *σ*, *f*, *ϕ*) from the set of cells in V1 with parameters sampled from the distribution of fits from the set of cells in MT (diagrammed in Fig 6A). We used the independent Poisson *FI* model for this analysis, and since the population *FI* distributions from V1 and V2 were quite similar, we restricted the analysis to V1 and MT. For each parameter, we first discarded the best-fit values from the V1 population. We then randomly sampled from a kernel-smoothed probability density derived from the set of best-fits to MT neurons and assigned new values to the parameter of interest for the V1 population, creating a hybrid population of Gabor tuning curves. As done previously, we then calculated the single cell *FI* distributions given this new set of tuning curves and summed their values to get the population *FI* (omitting negative values). We repeated this process 100 times to obtain the median and interquartile range for the hybrid populations. This process was then repeated for each of the Gabor parameters individually (Fig 6B). To quantitatively determine which resampled parameter produced an *FI* distribution that was closest to the empirical MT distribution, we computed the Jensen-Shannon (JS) divergence between each of the bootstrapped hybrid *FI* distributions and the empirical MT distribution (Fig 6C).

**Fig 7:**
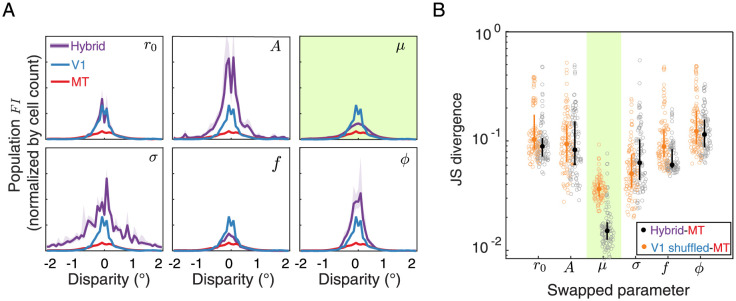
Control analysis for Gabor parameter resampling. **A**. The median and interquartile range of the population *FI* distributions from the 6 hybrid populations (purple) are plotted against the true V1 and MT population *FI* (same in each of the subplots). This panel is similar to Fig 6B except that the population *FI* is normalized by the cell count in each area rather than the total area under the curve. **B**. Jensen-Shannon (JS) divergence between the hybrid population *FI* distributions and the true MT population *FI* distribution are replotted from Fig 6C for each swapped parameter (black) along with the same quantities comparing the shuffled V1 population to the true MT population (orange). Lower values reflect a closer match between the two distributions. Open circles show the JS divergence between the population *FI* for each of the 100 hybrid bootstrapped populations; solid circles and errorbars show the median and interquartile range across all bootstraps.

We performed two control analyses to examine the generality of these results. First, we wanted to ensure that our focus on comparing population *FI* *shape* similarity instead of comparing the similarity in *area under the curve* (AUC) did not lead us to a false conclusion about the influence of each parameter. Therefore, we repeated our analysis without normalizing each bootstrap by the AUC and instead normalized solely by the number of cells in the bootstrapped sample. Overall, the results again show a notable *FI* similarity between the hybrid *μ* parameter populations and the true MT *FI* distribution (Fig 7A, top right plot), although the hybrid population created by resampling the frequency parameter also has an *FI* distribution shape similar to the shape of the true MT population. To ensure that the results of our resampling approach were due to resampling from the MT parameter distributions in particular and not due to simply shuffling the best fit V1 parameters, we also repeated our analysis by resampling the V1 parameter distribution from itself instead of from MT (i.e., we shuffled the parameters between V1 cells with replacement), and comparing this shuffled V1 population to MT (Fig 7B). Even when considering these control resamplings, the median JSD associated with applying the MT *μ* to the V1 population was still a factor of at least 2.4 lower than any others. These control analyses suggest that the specific changes in the distribution of preferred disparities in MT contributes to the change in *FI* distribution between V1 and MT.
